# Comprehensive, multidisciplinary care for fragile X-associated tremor/ataxia syndrome

**DOI:** 10.3389/fneur.2026.1746002

**Published:** 2026-03-19

**Authors:** James A. Bourgeois, Andrea Schneider, Jessica Klusek, Thomas R. Christensen, Ellie Levin, Kendall Gardner, Ariel A. Jacobi, Randi J. Hagerman

**Affiliations:** 1 UC Davis Health Department of Psychiatry and Behavioral Sciences, School of Medicine, Sacramento, CA, United States; 2 UC Davis Health MIND Institute and Department of Pediatrics, School of Medicine, Sacramento, CA, United States; 3 Department of Communication Sciences and Disorders, Arnold School of Public Health, University of South Carolina, Columbia, SC, United States; 4 UC Davis, Davis, CA, United States; 5 Touro University College of Osteopathic Medicine, Vallejo, CA, United States; 6 UC Davis School of Medicine, Sacramento, CA, United States

**Keywords:** fragile X syndrome, fragile X-associated tremor/ataxia syndrome, fronto-subcortical dementia/major neurocognitive disorder, FXAND, FXPOI, FXTAS

## Abstract

**Objective:**

The authors reviewed the pathophysiology, clinical genetics, phenotype, and comprehensive clinical management of Fragile X-associated Tremor/Ataxia Syndrome (FXTAS), a neurodegenerative disorder affecting *FMR1* premutation carriers (55–200 CCG repeats).

**Participants:**

A multispecialty and multidisciplinary team of authors with backgrounds in psychiatry, pediatrics, clinical psychology, speech and language pathology, and neurosciences.

**Evidence:**

Review of English language sources on the clinical phenomenology, genetics, pathophysiology, and clinical management of FXTAS from 2001 (the original report) through 2025, with emphasis on the general medical, psychiatric, and neurological features of FXTAS and its multispecialty and multidisciplinary clinical management.

**Consensus process:**

All authors contributed to the review of the literature. Major components of the manuscript were drafted by clinicians with clinical experience in the specific areas of management.

**Conclusion:**

Fragile X-associated Tremor/Ataxia Syndrome (FXTAS) is a neurodegenerative disorder affecting *FMR1* premutation carriers (55–200 CCG repeats) that is an illustrative model for a genetically-determined neuropsychiatric illness. It is characterized by intention tremor, gait ataxia, neurocognitive disorder, and other psychiatric symptoms. FXTAS occurs in patients with a family history of Fragile X-associated conditions, including Fragile X syndrome. Patients often develop fronto-subcortical dementia/major neurocognitive disorder (MNCD), leading to significant functional decline (particularly in males). A holistic, person-centered approach coordinated by the primary care clinician with multispecialty and multidisciplinary collaboration is recommended to address the complex needs of FXTAS patients and their support networks. This highlights the importance of integrated support for patients and caregivers, including addressing end-of-life/palliative care considerations.

## Introduction

**Figure 1:**
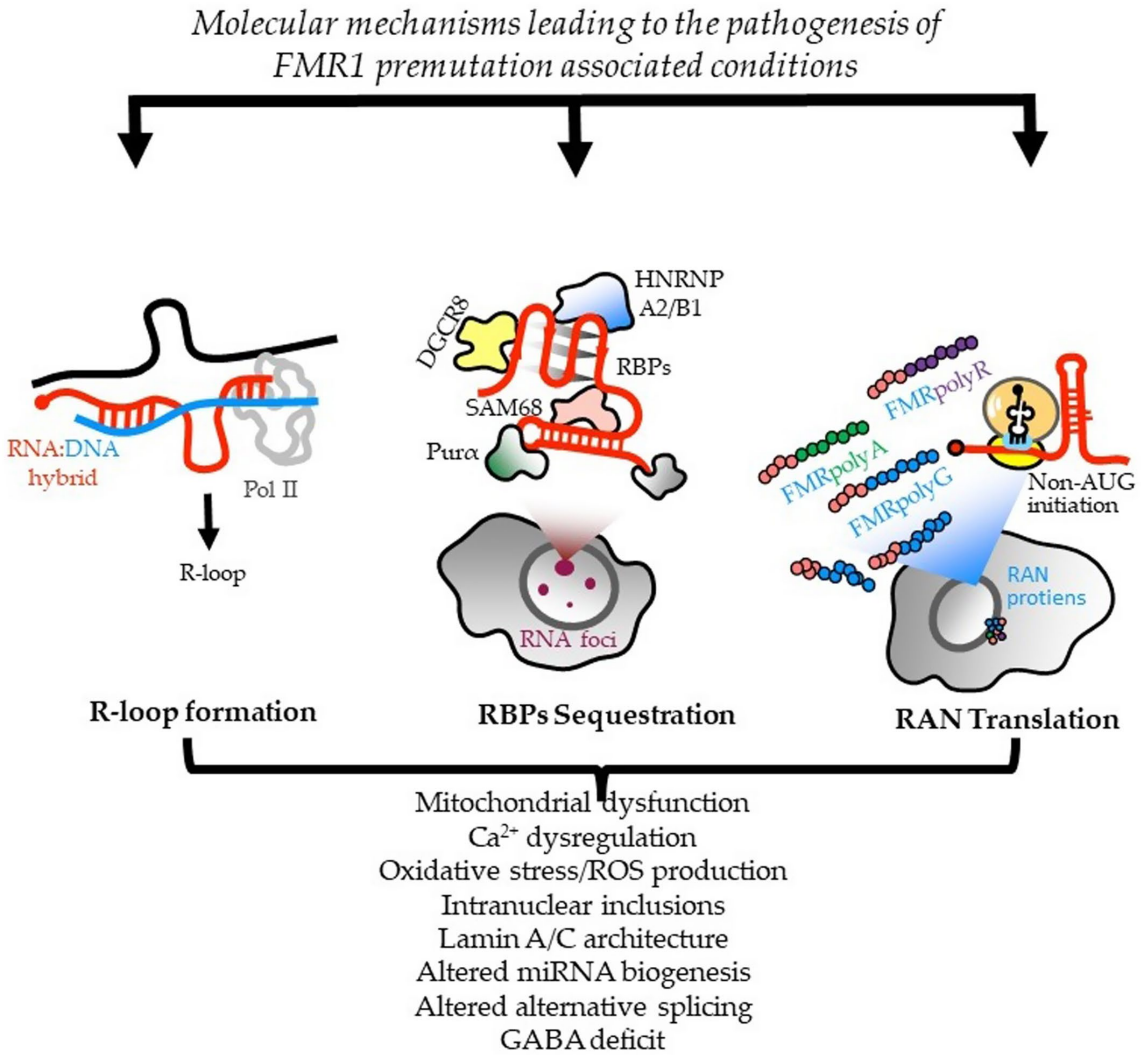
Molecular mechanisms leading to *FMR1*-PM-associated conditions. Three nonexclusive models are proposed for how CGG repeats contribute to the pathogenesis of PM conditions, including FXTAS. Reprinted with permission from Tassone et al. (8), licensed under CC BY.

FXTAS (pronounced *Fax*-tass) is a neurodegenerative disorder caused by a *premutation* [55 to 200 cytosine-guanine-guanine (CGG) repeats] in the Fragile X Messenger Ribonucleoprotein 1 (*FMR1*) gene on the X chromosome (Xq27.3), which was first reported in 2001 (1–3). Pathophysiologically, the premutation is associated with increased levels of *FMR1* mRNA, leading to toxic RNA gain-of-function effects on many systems, including decreased fragile X protein (FMRP), oxidative stress, mitochondrial dysfunction, calcium dysregulation, production of FMRpolyG thought to be toxic and sequestration of key proteins necessary for maintenance of neuronal health (2, 4–6). These changes result in the formation of eosinophilic ubiquitin-positive intranuclear inclusions in neurons and astrocytes and mitochondrial dysfunction (6, 7). This is the presumed mechanism contributing to central and peripheral nervous system pathology that is associated with its various neuropsychiatric clinical manifestations. The presumed multiple pathological pathways are illustrated in Figure 1 (8).

Generally, the higher the CGG repeat number in the premutation, the earlier the onset and greater the severity of symptoms (4). The prevalence of the premutation is estimated to be 1 in 148 to 200 females and 1 in 290 to 855 males in the general population. Males with the premutation have a lifetime risk of developing FXTAS that increases from 40% in their 60s to 75% in their 80s, whereas the risk for females has been less studied but is around 20% (9, 10). Males typically present with earlier onset and more rapid progression compared to females, with average ages of onset for tremor and ataxia occurring around 60 years for males, compared to around 70 years for females (5, 11–14).

## Relation to other fragile X-related illnesses

While our focus is on the clinical ascertainment and management of the FXTAS syndrome, it bears special emphasis to distinguish the *premutation carrier state* (leading to FXTAS) and other premutation conditions such as Fragile X-associated Premature Ovarian Insufficiency (FXPOI) and Fragile X-associated Neuropsychiatric Disorders (FXAND) from the *full mutation* state (1, 9, 11, 12, 15–20). The premutation state is limited to 55–200 CGG repeats. CGG repeats from 45 to 55 is considered a “gray zone” with varying degrees of neuropathology, while less than 45 is considered normal range. Many premutation carriers can be high functioning, thus “preclinical” in appearance and function into adulthood. It is presumed that the gradual accumulation of RNA toxicity throughout life can lead to neurodegeneration and other systemic signs later in adulthood.

The *full mutation* state, over 200 CGG repeats, leads to Fragile X syndrome (FXS). The full mutation state is characterized methylation of the gene and a lifelong deficiency in *FMR1* mRNA, hence inadequate *FMR1* protein (FMRP) production. FXS has a variable and often profound primary neuropsychiatric presentation in childhood, including high risk of intellectual disability (ID), autism spectrum disorders (ASD), anxiety disorders, and attention-deficit/hyperactivity disorder (ADHD) (21). FXS is the most common inherited form of ID and the most common single gene cause of ASD. With maternal transmission genetic amplification, the CGG repeat number increases when passed mother-to-child (22).

### FXAND

In young-middle adulthood, premutation patients generally do not experience cognitive impairment but have been found to have significantly higher rates of other psychiatric disorders, when compared to population norms, with as many as 50% of premutation carriers experiencing psychiatric illness antedating the motor symptoms characteristic of FXTAS (14, 19). These co-morbidities are referred to as *Fragile X-associated neuropsychiatric disorders* (FXAND) (6). These FXAND co-morbidities are primarily anxiety and depressive disorders (22). Bipolar II disorder is more common in premutation carriers compared to the general population, while bipolar I disorder is not (6). Psychotic disorders are not more common in premutation carriers than in the general population (6). Developmental delay, ADHD, autism spectrum disorder (ASD) and nonspecific aggression have increased prevalence in male premutation carriers, while developmental delay and ADHD are have increased prevalence in female carriers (3, 6, 23, 24). Female carriers have a higher rate of FXAND; these psychiatric symptoms may exacerbate some of their systemic illness symptoms (9). For manifest premutation-associated neuropsychiatric symptoms that do not lead to functional impairment, the term *Fragile X premutation-associated condition* (FXPAC) has been proposed as an alternative term (6, 23, 62).

The connection between anxiety and/or depressive disorders and the premutation carrier state may be overlooked or misattributed in clinical settings, unless clinical vigilance is increased based on known Fragile X-spectrum disorders in the patient’s pedigree. In many cases, the obligatory premutation carrier mother of a child (most commonly a son) with FXS may have experienced an anxiety and/or depressive disorder attributed solely to the social difficulties of raising an intellectually and behaviorally impaired child. However, premutation carriers have an increased risk of anxiety and depressive disorders, even if *not* raising a child with FXS. As such, it is more accurate to attribute anxiety and/or depressive disorders in premutation carriers to be a component of the premutation carrier condition itself (5).

### FXPOI

Fragile X-associated premature ovarian insufficiency (FXPOI) is commonly seen in premutation carriers and has been seen in patients as young as 11 (25). This can either be co-morbid with early motor and/or cognitive signs of FXTAS or in the absence of CNS symptoms (9). It is estimated to be present in approximately 10–20% of female premutation carriers and is characterized by early menopause (before age 40), often preceded by irregular menstrual function for more than four months (10, 23). The premutation is the most common single-gene cause of POI (23). Infertility is reported in up to 50% of premutation females. Curiously, some premutation carrier females with FXPOI do not later experience the motor and cognitive symptoms of FXTAS (26). FXPOI is associated with reduced life expectancy attributed to cardiovascular disease and osteoporosis attributable to abnormal female hormone function.

### FXTAS—clinical manifestations

The clinical manifestations of FXTAS include intention tremor, cerebellar ataxia, parkinsonian features, cognitive decline (in the phenotype of frontal-subcortical dementia/MNCD), and various psychiatric comorbidities including apathy, irritability, depressive disorders, and anxiety disorders (6, 9, 12, 15, 18, 24). Inclusions in pericardial ganglia and the cardiac conduction system are associated with arrhythmias (27). In male patients, erectile dysfunction is common. Dysphagia, choking, and constipation are also seen (27). Patients may also exhibit peripheral neuropathy and autonomic dysfunction, contributing to a decline in function in activities of daily living (ADLs). Given the progressive nature of FXTAS, patients often require steadily increasing levels of care, imposing substantial cumulative social emotional and physical burdens on caregivers (11, 12, 15–20).

Presumably because of the effect of cumulative RNA toxicity over decades, the early motor signs of FXTAS may emerge as early as in the 40s, but present more commonly in the 60s. Initial signs include tremor, which is more prominent in the distal upper extremities. This can lead to diminution of fine motor control for manual tasks, such as writing, eating, and using tools or instruments. With progression, cerebellar ataxia (with gait and station problems) develops, causing frequent falls, affecting mobility or even the ability to maintain a standing posture. Patients may become increasingly reliant on assistive devices (first a cane, later a walker) to cope with motor impairments. Because of the age of onset with prominent tremor, which can include a resting tremor, these patients are often misattributed as having Parkinson’s disease, multiple sclerosis, or other neurological illness. The progression of FXTAS follows a stereotypical pattern, with stages that guide therapeutic interventions (11–13, 15–20, 24). The diagnostic criteria for FXTAS are summarized on Table 1, and the stages of progression of FXTAS are summarized on Table 2 (14, 28, 29).

**Table 1:** Diagnostic criteria for FXTAS (14, 28, 29).

Definite: Presence of 1 major radiological sign plus 1 major clinical symptom
Probable: Presence of either 1 major radiological sign plus 1 minor clinical symptom or 2 major clinical symptoms.
Possible: Presence of 1 minor radiological sign plus 1 major clinical symptom
Radiological Signs
Major: MRI white matter lesions in MCPs and/or brain stem
Minor: MRI white matter lesions in cerebral white matter
Minor: Moderate to severe generalized atrophy
Minor: MRI white matter lesions in the splenium of the corpus callosum
Clinical Symptoms
Major: intention tremor
Major: Gait ataxia
Minor: Parkinsonism
Minor: Moderate to severe short-term memory deficiency
Minor: Executive function deficit
Minor: Neuropathy in lower extremities
Neuropathology
Major: FXTAS intranuclear eosinophilic inclusions that are ubiquitin or p62-positive

**Table 2:** Stages of FXTAS progression.

Stage 1: Subtle or questionable tremor and balance problems may be observed. Symptoms might be overlooked as “benign aging” signs.
Stage 2: Mild tremor and balance problems become evident but have limited interference with ADLs. Patients remain largely independent.
Stage 3: More pronounced tremor and balance problems lead to significant difficulties in ADLs. At this stage, motor symptoms restrict mobility and functional independence.
Stage 4: Severe tremor and ataxia necessitate assistive devices such as a cane or walker. Dependency on caregivers increases as ADLs are increasingly impaired.
Stage 5: Patients become wheelchair users, with limited ability to ambulate independently.
Stage 6: At the final stage, patients are bedridden and require comprehensive care for all ADLs, with profound motor and cognitive impairments.

Usually following the movement disorder by a variable number of years, patients can develop a frontal-subcortical dementia/MNCD, affecting 50% of males with FXTAS (13). This is a dementia syndrome with progressive impairment of cognitive function with both frontal lobe and subcortical features. The *frontal lobe* impairment (like what is seen in frontal-temporal dementia) includes dysexecutive function, with prominent disinhibition (e.g., behavioral dyscontrol, impulsive, inappropriate social behavior) (22, 24). Information processing speed is commonly affected in FXTAS, particularly in male subjects (24). Affective signs associated with frontal lobe impairment include anxiety, hostility, apathy, and irritability (7). The *subcortical* signs include concurrent movement disorder, mood symptoms, apathy, and slowing in speech and thinking, as is seen in Parkinson’s disease and Huntington’s disease. Decreased executive function may correlate with greater motor impairment (10, 17, 27).

It is common in early FXTAS dementia/MNCD to see relative preservation of other cortical functions such as speech, thought organization, orientation, judgment, recognition, problem solving, and naming. Specific cognitive domains impacted in FXTAS dementia/MNCD include attention, visuospatial processing, executive function, and memory (24). The co-occurrence of frontal lobe and cerebellar signs in FXTAS is consistent with abnormalities of cerebro-cerebellar circuits (2). Given the tri-morbidities of motor, cognitive, and anxiety/depressive symptoms commonly seen, it has been plausibly hypothesized that FXTAS ultimately produces damage to cortico-ponto-cerebello-thalamo-prefronto-cortical loops (2). The concurrence of abnormal executive function, impaired visuospatial and speech functions, and emotional blunting or disinhibition has been described as the cerebellar cognitive affective syndrome (CCAS) (2).

The onset of FXTAS typically occurs in premutation carriers over the age of 60, with men being more severely affected than women. The greater risk and more malignant course of illness in males can be attributed to the fact that premutation females benefit from the presence of an intact other X chromosome, which mitigates some FXTAS risk and severity. Females have high rates of hypothyroidism, while males have a more rapid disease progression (7, 25, 30).

Particularly in female patients, FXTAS may bear some overlap with the motor signs of multiple sclerosis (which is seen in about 3% of FXTAS patients). Female patients have a high risk of numerous other systemic illnesses. These include autoimmune disease, hypertension, sleep disorders, osteoporosis, as well as neurologic symptoms of neuropathy, migraine, chronic pain/fibromyalgia, bladder dysfunction, olfactory dysfunction, abnormalities of ocular movements, and vestibular disorders (9, 13, 14, 22, 23, 25, 26). Increased age, smoking, increased BMI, and depressive disorder are strongly associated with several of the systemic illnesses seen in premutation women (22). Appropriate clinical vigilance for the clinical context of presentation may assist the clinician. One also needs to be mindful of the possibility of FXTAS comorbid with other illnesses with neurological and psychiatric signs and symptoms, as well as more obscure illnesses with similar phenotypic presentation (31–33).

Although FXTAS is a progressive neurodegenerative disorder there is typically significant abrupt dysfunction that can occur with systemic infectious disease. Often these are sudden onset episodes of severe weakness and lack of ability to walk that are related to infection such as a urinary tract infection or pneumonia. However, two cases have been reported of paroxysmal episodes of cerebellar and/or brainstem dysfunction that occurred abruptly with in patients with FXTAS, however the etiology for these episodes was not found (34, 35).

Astrocytes appear to be the most vulnerable to the effects of RNA toxicity and they die first in FXTAS (36). Astrocyte death is followed by neuronal death, leading to the WMH. Exposure to toxins such as excessive alcohol and/or opioids can worsen WMH (37). Sleep apnea and hypoxia can worsen WMH, so it is important to have a sleep apnea study, since sleep apnea is common in FXTAS. If a sleep apnea study is positive, then CPAP is indicated. Exercise can lower inflammation and improve mitochondrial function, so daily exercise is warranted; this can be guided by a physical therapist/PT. Since ambulation can be problematic in late stage FXTAS, using a stationary exercise bicycle with a recombinant seat for exercise can help to reduce the risk of falling.

## Differential diagnosis

Patients presenting in mid-life with FXTAS need to be differentiated from other causes of acquired movement disorder (38). Inasmuch as the presentation of FXTAS can be variable, this distinction can be challenging. Based on its much higher prevalence, the primary differentiation of FXTAS is from Parkinson’s disease (39). FXTAS may also resemble other hereditary ataxias, spinocerebellar atrophy, multiple system atrophy, Alzheimer’s disease, fronto-temporal dementia, restless legs syndrome, dysautonomia, and essential tremor (9, 18, 39–42). In FXTAS, neurons from the substantia nigra can also die, such that about 30% of FXTAS patients can have parkinsonian symptoms, such as resting tremor, flat facies, and shuffling gait. CNS pathology studies have shown that 4% can have both FXTAS inclusions and the Lewy body inclusions of Parkinson’s disease (39).

There are very similar clinical features of neuronal intranuclear inclusion disease (NIID) and FXTAS in terms of tremor, ataxia, cognitive decline and MRI findings. However, the intranuclear inclusions of FXTAS are not seen in the fibroblasts, whereas this is a typical place for NIID inclusions. NIID is most seen in Asian populations, whereas FXTAS is present in all populations. To clearly distinguish between the two disorders DNA testing will reveal the expansion in different genes, specifically the *NOTCH2NLC* gene in NIID and *FMR1* in FXTAS (43).

### Pedigree

When FXTAS is suspected, the patient’s multigenerational pedigree should be assessed. The full mutation Fragile X syndrome can affect both boys and girls, but penetrance is higher in boys due to the X-linked nature of the condition, and expressivity of the phenotype is much more variable in girls. The phenotype of Fragile X syndrome ranges from subtle to severely impairing, but commonly include intellectual disability, developmental delay, speech abnormalities, autistic social behaviors, and physical findings such as macroorchidism, large ears, and prominent chin.

The mothers of Fragile X males are obligate premutation carriers (unless they have the full mutation) and may have variable clinical manifestations, including Fragile X-associated primary ovarian insufficiency (FXPOI), thyroid disease, autoimmune disorders, and anxiety and depressive disorders. The fathers of carrier females (the grandfathers of Fragile X males) are often premutation carriers (although the premutation can come from premutation females to their daughters). They also have an elevated risk of anxiety and depressive disorders in adulthood, antedating the motor and cognitive symptoms of the FXTAS phenotype. In cases where a male FXTAS patient does not have any daughters (hence no Fragile X illnesses in his descendants), assessment of family members is important because his other relatives may be affected, since his mother had been an obligate carrier. Due to genetic anticipation, the CGG repeat length expands from premutation to full mutation when passed from (premutation) mother to (full mutation) son (25).

### Genetic counseling

Genetic counseling serves as a critical intervention to identify at-risk relatives, facilitate cascade testing, and support reproductive decision-making. Maternal transmission of premutation alleles which includes amplification of the CGG repeat number poses a risk for Fragile X syndrome in descendants, while both male and female relatives may unknowingly carry premutation alleles with potential clinical significance. Genetic counselors can help families navigate the emotional impact of learning about carrier status, address concerns about illness-related stigma, and provide education about inheritance patterns, recurrence risk, and available testing options. Referrals to genetic counseling should be prioritized at the time of FXTAS diagnosis, with encouragement to involve family members in counseling sessions as appropriate (with patient consent). Such interventions not only clarify genetic risks but also empower patients and their families to make informed medical and reproductive decisions.

### Clinical evaluation

At typical FXTAS onset, patients will present with the motor findings (particularly cerebellar ataxia and/or intention tremor). Memory and executive function deficits are also common (17, 19). When there is any suspicion of FXTAS based on presentation and/or pedigree analysis, genetic testing for the Fragile X premutation by Fragile X DNA testing will reveal the CGG repeat number in the *FMR1* gene. This will confirm the diagnosis of the premutation. Given the specificity of the genetic testing for FXTAS, it would be reasonable to routinely assess all new-onset tremor and/or ataxia patients with Fragile X DNA testing. The motor symptoms are major criteria for the diagnosis of FXTAS, but an MRI is recommended to evaluate for the middle cerebellar peduncle (MCP) sign, hyperintensity and thinning of the corpus callosum, brain atrophy and other neuroanatomic abnormalities seen in FXTAS that are less specific (16, 18).

**Figure 2:**
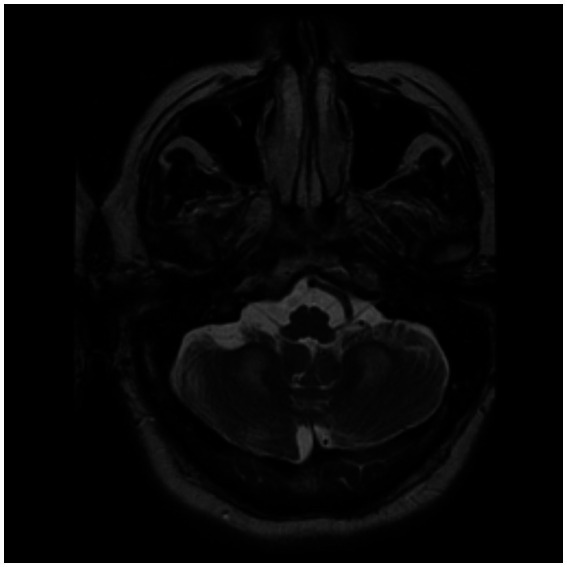
MRI of hyperintensities of the middle cerebellar peduncles (MCP).

MRI of the brain with particular attention to the hyperintensities in the MCPs should be accomplished (16, 18). The volume of periventricular and supratentorial white matter hyperintensities (WMH) correlates with progression of FXTAS, cognitive decline, and severity of motor symptoms (27, 44). Hyperintensities in the MCPs are seen in 60% of male FXTAS patients and is considered highly specific for FXTAS in the appropriate clinical context; the MCPs also exhibit thinning as the disease progresses (6, 10, 16–18, 31, 45, 46). The abnormalities in the MCPs may be the pathway linking the cortex to the cerebellum and thus may account for the co-morbid cognitive and movement disorder symptoms (10). In females with FXTAS there is typically involvement with WMH in the splenium of the corpus callosum (which can also exhibit thinning) (9). High-intensity signal at the corticomedullary junction has also been reported in some cases (47). Cortical atrophy and ventriculomegaly are also common, especially at advanced stages (9, 45, 46). With illness progression, volume loss in whole brain, cerebellum, brain stem are seen, as are increases in WMH (44) (Figure 2).

In the case where a patient presents with cognitive and other psychiatric symptoms associated with motor signs, a “dementia with a movement disorder” assessment should be done, with considerations of Parkinson’s disease, Lewy body dementia, fronto-temporal dementia, multiple sclerosis, spinocerebellar ataxias, and other phenotypically similar conditions. Given the lack of specificity in many dementia evaluations, we recommend considering Fragile X DNA testing in all new cases of dementia/MNCD with a movement disorder, even in cases without the classic Fragile X disorders inheritance pattern in family members. Pedigree analysis seeking the inheritance of Fragile X-associated illnesses in family members may help to focus the work-up. It would be reasonable, though of far lower yield, to order Fragile X DNA testing in all new cases of dementia/MNCD.

### Current gaps in research and treatment

While significant advances have been made in understanding the molecular and clinical aspects of FXTAS, research gaps persist, particularly regarding disease-modifying therapies, psychiatric outcomes, and support for caregivers (44). Current research addresses the ascertainment of rate of progression of CNS symptoms, the role of medications and supplements (e.g., antioxidants, cardiovascular medications) in disease progression, and disease-modifying interventions.

### Holistic care components for FXTAS patients

Since FXTAS is usually a slow yet inexorably progressive neuropsychiatric illness, comprehensive ongoing outpatient care is needed (48). This care team is best led and coordinated by a primary care physician with ready ongoing access to medical and other health professional consultants for co-management. Specific consultants typically involved in the care of FXTAS patients include psychiatrists, neurologists, clinical psychologists, speech/language pathologists, physical therapists, occupational therapists, and social workers. The ongoing collaboration among neurology, psychiatry, and clinical psychology is particularly desirable. The absence of tailored care models addressing general medical, neurological, and psychiatric illness in FXTAS underscores the need for a comprehensive, multispecialty, multidisciplinary approach to FXTAS management. We outline a holistic care strategy integrating medical, psychological, and rehabilitative interventions to optimize the quality of life for patients and their families. As there are no gene-specific interventions to change disease course for FXTAS at present, clinical interventions to date focus on symptom management and secondary prevention. Many of these interventions overlap with overall preventive health care but are of particular importance in neurodegenerative illness.

## General medical management

To mitigate the progressive symptoms and impairment from FXTAS, primary care physicians need to take an assertive stance on health promotion. These include avoidance of smoking, alcohol consumption, and drug abuse. This is particularly important when patients develop significant tremor and ataxia. To a degree compatible with safety (being mindful of the patient’s motor status), regular cardiovascular exercise is highly recommended. Primary care clinicians should take an aggressive stance in the medical management of hypertension, hyperlipidemia, diabetes mellitus, and obesity. Optimized management of cardiovascular risk factors could help to minimize the risk of vascular cognitive impairment. For patients with history of FXPOI, hormone replacement therapy is indicated to mitigate cardiovascular risk and prevent osteoporosis (36). Correction of vitamin deficiencies and hypothyroidism is essential. Antioxidants (e.g., alpha-tocopherol, coenzyme Q19), cannabidiol (CBD), gabapentin and anti-inflammatory medications are considered for chronic pain management (19, 23). FXTAS patients suffer from frequent infections because the immune system is impaired from ongoing RNA toxicity. A simple flulike illness, urinary tract infection or COVID can lead to deterioration and need for hospitalization. The most frequent infection is recurrent urinary tract infections (UTIs) that may not respond well to antibiotics, and in these cases the Uqora^1^ system can be helpful to lower the risk of recurrent UTIs. There have been no studies focused specifically on most of the antioxidants, CBD and Uqora except for the study of sulforaphane. an antioxidant (49). However, some patients have found benefit from Uqora system, and overall, these recommendations are based on data from other neurodegenerative disorders.

Caution is needed to minimize delirium risk, especially when these patients are hospitalized (13, 17). They may be particularly vulnerable to cancer chemotherapeutics and general anesthesia, particularly isoflurane (50). After these patients develop fronto-subcortical dementia/MNCD, they should be managed as “delirium-prone” patients. This includes avoidance of medications well-known to increase delirium risk and/or severity (e.g., benzodiazepines, Z-drugs, anticholinergics, opioids, antihistamines, anesthetics, stimulants, corticosteroids). Doses of CNS-active medications should be started at lower-than-typical doses and cautiously titrated, due to the presumed abnormal function of the vulnerable blood–brain barrier in neurodegenerative disease, which increases the risk of CNS side effects. Patients should minimize or eliminate use of alcohol, which can exacerbate cognitive impairment and have directly toxic effects on cerebellar function, leading to further impairments in motor function (25).

### Neurologic symptom management

While there is no disease-modifying intervention for FXTAS, active involvement of a movement disorder neurology consultant is recommended. Pharmacological interventions that target tremor in FXTAS include primidone, levetiracetam, gabapentin, pregabalin, and beta-adrenergic blockers (9, 19). When parkinsonian features are present, carbidopa/levodopa can be considered, but with a low threshold to modify or discontinue treatment if there is not meaningful symptom improvement, or if there are problematic medication-specific side effects (e.g., psychotic symptoms). Allopregnanolone, which may have neuroprotective effects for the mitochondria, may be considered for motor and sensory symptoms (9, 17, 19).

### Psychiatric symptom management

As with many other neuropsychiatric illnesses, simultaneous management of motor as well as psychiatric symptoms is often necessary in FXTAS. Regular monitoring of cognitive status with standardized cognitive rating scales (e.g., Montreal Cognitive Assessment (MoCA), Mini-Mental State Examination (MMSE)) is necessary to monitor the rate of progression of cognitive symptoms. Depressive and anxiety disorders are common before onset of motor signs and may persist or worsen afterwards. Aggressive treatment of anxiety and depressive disorders may improve cognitive function in FXTAS.

Following a diagnostic interview, supported by psychiatric symptom rating scales, antidepressants are often indicated. *Selective serotonin reuptake inhibitors* (SSRIs) are a common first antidepressant choice but can be associated with transient overstimulation, GI distress, and sexual dysfunction. Clinicians should pay particular attention to other risks associated with SSRIs in medically vulnerable patients. Drug–drug interactions with multiple other medications are problematic with fluoxetine, paroxetine, and fluvoxamine; as such, the preferred SSRIs in patients with other chronic illnesses are sertraline, citalopram, and escitalopram. SSRIs increase the risk of bleeding and should be avoided in thrombocytopenia and other bleeding-prone states (e.g., hemorrhagic stroke, GI bleed). All serotonergic psychotropic medications have a risk of SIADH (syndrome of inappropriate antidiuretic hormone = hyponatremia in an euvolemic state with low serum osmolality and high urine osmolality), may present with delirium. Among psychotropics, SSRIs have the highest SIADH risk. *Serotonin-norepinephrine reuptake inhibitors* (SNRIs) have a similar mechanism of action to SSRIs and are subject to the same cautions. However, attributable to their noradrenergic activity, they can also help to manage neuropathic pain (9). *Dopamine-norepinephrine reuptake inhibitors* (DNRIs, of which bupropion is the only one available in the US) have a similar mechanism of action. Bupropion is the preferred antidepressant for depressed patients with hypersomnia and weight gain, but in FXTAS, its stimulating effect could increase tremor, so it must be used cautiously. Patients with melancholic depression (with prominent insomnia and lack of appetite) may be started on the *atypical antidepressant* mirtazapine (which promptly promotes sleep and appetite). For treatment-resistant depressive disorder, advanced interventions such as antidepressant augmentation, esketamine, ketamine, transcranial magnetic stimulation (TMS), and electroconvulsive therapy (ECT) can be considered following inadequate response to medication optimization (9, 22).

Antidepressants are also the first-line pharmacological intervention for most anxiety disorders. The *partial 5-HT1A agonist* buspirone is a non-benzodiazepine (BZP) anxiolytic that can improve symptoms of generalized anxiety disorder and can be safely combined with antidepressants. Due to their associated risk for dependence, motor impairments, and cognitive vulnerability, benzodiazepines and z-drugs should be avoided in FXTAS, as they are avoided in other MNCD/dementia syndromes.

Mood stabilization with *anticonvulsants and/or second generation antipsychotics* can be helpful for patients with history of bipolar disorder and/or with significant irritability/impulsivity associated with FXTAS dementia/MNCD. Anticonvulsant risks include hyperammonemia, hepatotoxicity, and pancreatitis with valproate; hematologic and hepatic side effects with carbamazepine, SIADH risk with valproate, carbamazepine, and oxcarbazepine, and toxic epidermal necrolysis (TEN) and Stevens-Johnson syndrome with lamotrigine. Due to its systemic medical risks and risk of tremor, lithium is usually avoided.

*Second generation antipsychotics* have increased morbidity and mortality risk in dementia/MNCD, thus should not be used nonspecifically for agitation in FXTAS dementia/MNCD. These medications, at low doses with close monitoring, are used for the specific psychotic symptoms (e.g., delusions, hallucinations) infrequently seen in FXTAS for psychotic symptoms, due to its lower level of dopamine receptor blockade compared to other second generation antipsychotics. Quetiapine is the preferred antipsychotic in patients with concurrent movement disorders (25). Anticholinergic agents are avoided, due to their risk of increasing cognitive decline, GI dysmotility, and delirium risk (25).

Patients with major depressive disorder refractory to optimized medication management should be referred to a neuromodulation psychiatrist. Currently FDA approved neuromodulation techniques for treatment-resistant depressive disorder include intranasal esketamine, repetitive transcranial magnetic stimulation (rTMS), vagal nerve stimulation, and electroconvulsive therapy (ECT). The neurosurgical procedure of deep brain stimulation (DBS) as is commonly used for tremor and Parkinson’s disease is undergoing clinical trials for treatment-resistant depressive disorder. IV ketamine, off-label, is also used for major depressive disorder.

Conventional dementia/MNCD treatment with acetylcholinesterase inhibitors and memantine (FDA approved for Alzheimer’s disease) can be tried for cognitive symptoms in FXTAS. These medications are unlikely to *reverse* cognitive impairments but could *slow the rate* of anticipated cognitive decline. Acetylcholinesterase inhibitors are held for pulse < 60 BPM. A previous controlled trial of memantine in FXTAS did not improve tremor or ataxia (51). However, a memantine controlled trial did improve measures of memory and auditory processing on event related potentials (ERP) studies (52, 53).

### Cognitive stimulation and digital brain training

Non-medical interventions, such as cognitive training programs, offer a promising adjunct to traditional care for patients with FXTAS, particularly in the early stages when cognitive reserve is relatively preserved. Digital platforms like BrainHQ, CogniFit, and Lumosity provide “gamified” exercises targeting executive functioning, memory, attention, and processing speed.

Systematic reviews and meta-analyses have demonstrated modest, but meaningful, improvements in cognitive outcomes among older adults and patients with mild cognitive impairment when engaging in digital cognitive training (54, 55). These gains appear strong when cognitive training is combined with physical exercise and personalized engagement strategies. While data specific to FXTAS-related dementia/MNCD are lacking, the low cost and accessibility of these tools support their inclusion as a low-risk intervention. Prospective study of these techniques for cognitive preservation would be desirable in a FXTAS dementia/MNCD population. Clinician-guided selection and monitoring of these programs, ideally with input from neuropsychologists or occupational therapists, may optimize their clinical utility. Programs that blend real-world cognitive demands (e.g., organizing tasks, memory journaling) with app-based games may offer the best generalization of training effects to everyday life. More research is needed to assess the direct impact of these tools in the FXTAS population, but their low cost and accessibility support their inclusion in a comprehensive care model.

### Psychotherapy—for patients and caregivers

Caregivers experience high levels of stress due to the increasing demands of caring for a person with FXTAS. The emotional and physical toll of caregiving can lead to burnout, psychiatric symptoms, and diminished quality of life. This is a pattern that has been noted in caretakers for patients with other dementia/MNCD syndromes, such as Alzheimer’s disease. Often the FXTAS patient’s caregiver is significantly depressed also, sometimes with worse psychiatric symptoms than the FXTAS patient. Psychiatric consultation, psychotherapy, and psychopharmacological intervention for caregivers also should be considered in addition to psychotherapy (55).

Psychotherapy interventions play a critical role in addressing the emotional, relational, and existential challenges faced by individuals with FXTAS and their caregivers. Aging patients affected by neurodegeneration often experience grief over functional loss, identity shifts, increased dependency, and social isolation. Concurrently, caregivers face elevated risks of depression, anxiety, and caregiver burden, which may surpass the psychological distress experienced by the patient. Individual psychotherapy for patients can focus on adjustment to illness, grief processing, and maintenance of self-efficacy despite disease progression. Cognitive-behavioral therapy (CBT), acceptance and commitment therapy (ACT), and existential therapies have demonstrated efficacy in promoting psychological adaptation in chronic neurodegenerative conditions (56, 57).

For caregivers, interventions such as cognitive-behavioral stress management, mindfulness-based stress reduction (MBSR), and psychoeducation have been shown to reduce psychological distress and enhance coping (58). Family therapy or dyadic counseling can help improve communication and mutual support between patients and caregivers. Integration of psychotherapy into the multidisciplinary FXTAS care model acknowledges the emotional dimensions of living with and caring for someone with neurodegeneration, ultimately promoting resilience and quality of life for both parties. Structured workshops focusing on stress relief techniques (e.g., mindfulness, meditation, and relaxation exercises) can significantly reduce caregiver burden and enhance coping mechanisms.

### Social support networks

Peer support groups for both patients and caregivers provide a platform for sharing experiences, fostering a sense of community, and enhancing psychological well-being. These networks can be accessed with a social worker who is aware of local clinical resources. Participation in the local senior center can provide important social activities.

### Management of swallowing disorders (dysphagia)

FXTAS patients who complain of frequent choking, sensation of food getting stuck, or coughing while eating should be referred to a speech and language pathologist (SLP) for a swallowing evaluation. When left unmanaged, swallowing disorders (i.e., dysphagia) can lead to weight loss, dehydration, and aspiration pneumonia, and are associated with increased risk of hospitalization, morbidity, and mortality. The assessment of dysphagia may include both clinical and instrumental procedures, such as a modified barium swallow study (MBSS), which will allow the SLP to evaluate underlying structural and physiological problems of the oral cavity, pharynx, larynx, upper esophagus, and the respiratory system that may be contributing to the abnormal swallowing. Diagnostic workup may require input from gastroenterology when the signs/symptoms of dysphagia suggest an esophageal etiology such as esophageal dysmotility or gastroesophageal reflux disease (GERD). The treatment of dysphagia is tailored toward the patient’s specific needs and the underlying cause of swallowing difficulty; therefore, referral to SLP for comprehensive dysphagia assessment is essential to ensure that clinical management recommendations are appropriate and maximally effective (Table 3).

**Table 3:** Recommendations for dysphagia management.

1. Positional strategies: Postural adjustments such as tucking the chin or tilting the head as well as maintaining upright posture can increase control of swallowing by redirecting bolus flow.
2. Swallowing maneuvers: Techniques to modify the strength or timing of swallowing movement may be recommended such as effortful swallow supraglottic swallow or the Mendelsohn maneuver.
3. Diet texture modifications: Changes in diet texture or consistency such as thickening liquids or pureeing softening or chopping solids can improve bolus control.
4. Behavioral strategies: Slower pacing taking smaller sips/bites and alternating solids may be recommended. Some patients benefit from supervised feeding to monitor use of compensatory strategies.
5. Direct rehabilitation: Biofeedback neuromuscular electrical stimulation and swallowing exercises may be used to target strength coordination range of motion and proprioceptive feedback of the muscles involved in swallowing.

### Management of vocal dysfunction and slow, slurred, or imprecise speech

SLPs can diagnose and treat dysphonia (voice problems) and dysarthria (speech problems) that arise in FXTAS patients. The most common voice problems seen in FXTAS include vocal tremor and spasmodic dysphonia, which can present clinically as a shaky, hoarse, or tight voice (59, 60). Voice assessments are typically conducted by an SLP in collaboration with ENT and may include stroboscopy to visualize the movement of the vocal folds and an assessment of vocal quality. Management of voice disorders may involve behavioral voice therapy provided by an SLP as well as physician-managed pharmacological interventions, depending on the underlying cause. Slow, slurred, or imprecise speech in patients with FXTAS may be due to dysarthria, a motor speech disorder. Dysarthria can hinder effective communication and negatively impact social interactions, so patients with reduced speech intelligibility should be referred to SLP for evaluation and treatment. Treatment strategies for dysarthria include enhancing control and coordination of the lips and tongue articulator, improving respiratory support to increase speech clarity and volume, and incorporating alternative forms of communication such as gestures, writing, communication books, or assistive technology.

### Management of cognitive-communication deficits

SLPs can play a critical role in supporting patients with FXTAS and their caregivers by addressing communication and interpersonal difficulties resulting from the cognitive deficits that can occur, especially in later stages of disease. MNCD/dementia and behavioral disinhibition can contribute to disorientation, reduced emotional connection, safety concerns, and challenging behaviors—factors that significantly increase burden on caregivers. SLPs can help mitigate these challenges by working collaboratively with patients and caregivers to implement compensatory strategies. Examples of compensatory strategies for cognitively impaired patients include establishing predictable routines and using external memory aids (e.g., signs, labels) to enhance orientation, reducing clutter and background noise to improve attention, using external aids such as planners, checklists, and alarms to support memory and participation in daily activities, and appropriate assistive technology to support cognitive function and communication.

### Audiology

FXTAS can impact cranial nerve (CN) VIII, leading to sensorineural hearing loss and/or tinnitus. Older adults with untreated hearing loss are at heightened risk for social isolation, depressive disorders, and acceleration of cognitive decline in MNCD/dementia. Timely referral to audiology for hearing evaluation and consideration of hearing aids or other assistive devices is a critical component of care (61).

### Physical therapy

Physical therapy (PT) is a cornerstone in managing motor symptoms associated with FXTAS. Tailored interventions focusing on balance and strength training are essential to mitigate fall risks and enhance mobility. Balance training exercises, such as static and dynamic postural control activities, have been shown to improve gait stability and reduce the incidence of falls in patients with ataxia-related disorders. Incorporating strength training, particularly targeting lower limb and core muscles, can further support postural stability and functional independence. Aquatic therapy offers a low-impact environment conducive to improving balance and strength, making it a valuable modality for patients with FXTAS.

### Occupational therapy

Occupational therapy (OT) plays a pivotal role in assisting patients with FXTAS to maintain independence in activities of daily living (ADLs). Therapists assess the patient’s functional abilities and recommend adaptive strategies and assistive devices to facilitate daily tasks. For instance, the use of weighted utensils can help counteract tremors during meals, while dressing aids like button hooks can simplify clothing management. Environmental modifications, such as installing grab bars in bathrooms and removing tripping hazards, are critical to creating a safe living space and preventing falls. Occupational therapists also provide training on energy conservation techniques and task simplification to accommodate the patient’s evolving capabilities.

### Continuous patient and caregiver education

Education is fundamental in empowering patients with FXTAS and their caregivers to manage the disease optimally. Providing comprehensive information about the progression of FXTAS, symptom management, and available resources enables informed decision-making and proactive care planning. Engagement with organizations such as the National Fragile X Foundation (NFXF: https://fragilex.org) offers access to educational materials, support networks, and updates on ongoing research. Caregivers benefit from education on coping strategies, stress management, and respite care options, which are vital in mitigating caregiver burden and promoting well-being. Encouraging participation in support groups, even those not specific to FXTAS, can provide valuable emotional support and shared experiences.

### Tailored support interventions

Caregivers can receive regular assessments to track well-being and identify early signs of burnout. Counseling and support groups run by mental health professionals and peer networks offer emotional support and practical advice. Respite care services can provide temporary relief for caregivers to rest and focus on their own well-being (Table 4).

**Table 4:** Resources for support of FXTAS patients.

National Ataxia Foundation. (n.d.). 11 Exercises for Ataxia Patients. Retrieved from https://www.ataxia.org/11-exercises-for-ataxia-patients/NationalAtaxiaFoundation+3NationalAtaxiaFoundation+3NationalAtaxiaFoundation+3
Melo RS, Cardeira CSF, Rezende DSA, Guimarães-do-Carmo VJ, Lemos A. Effectiveness of aquatic physical therapy exercises to improve balance, gait, quality of life and reduce fall-related outcomes in healthy community-dwelling older adults: A systematic review and meta-analysis. PLOS ONE 2023; doi:10.1371/journal.pone.0289555Wikipedia
National Institute of Child Health and Human Development. (n.d.). What are some types of assistive devices and how are they used? Retrieved from https://www.nichd.nih.gov/health/topics/rehabtech/conditioninfo/deviceNICHD
American Occupational Therapy Association. (2024). Increasing participation with home modifications across the lifespan. Retrieved from https://www.aota.org/publications/sis-quarterly/home-community-health-sis/hchsis-11-24AOTA
National Fragile X Foundation. (n.d.). I Have FXTAS: Now What? Retrieved from https://fragilex.org/premutation/fxtas/i-have-fxtas-now-what/NationalFragileXFoundation+1NationalFragileXFoundation+1
National Fragile X Foundation. (n.d.). FXTAS Caregivers: There is Help! Retrieved from https://fragilex.org/premutation/fxtas/fxtas-caregivers-there-is-help/

### End-of-life care in FXTAS: palliative and hospice care

As FXTAS is an inexorably progressive illness with continuous loss of function and independence, a holistic approach must sensitively address end-of-life management. As FXTAS progresses to the later stages, palliative care focuses on symptom management, comfort, and quality of life. Symptom relief includes addressing physical discomfort, including pain, mood symptoms, and sleep problems. Emotional and psychological support can include access to grief-focused counseling for patients and families to manage the emotional aspects of end-of-life care.

### Planning and advanced directives

Care planning involves coordination of healthcare services, establishment of care preferences, and decision-making about hospice care options to align with the patient’s and family’s wishes. This should be done early in the course of illness, before dementia/MNCD progression impacts the patient’s decisional capacity to agree to an advance care plan and goals of care discussions. Due to the progression of cognitive impairment, ascertainment of the patient’s decisional capacity for care planning should be accomplished early in the course of illness. Early initiation of advance care planning (ACP) ensures that the patient’s values and care preferences are documented before significant cognitive impairment limits participation. ACP conversations should ideally begin when patients are in Stage 4 of FXTAS progression where a walker or wheelchair is needed.

Evidence-based tools such as PREPARE for Your Care,^2^ Five Wishes,^3^ and The Conversation Project^4^ can guide patients and families through structured discussions about medical goals, quality of life priorities, and end-of-life preferences. Clinicians should encourage documentation of advance directives, durable power of attorney for health care, and POLST (Physician Orders for Life-Sustaining Treatment) forms where applicable. Integration of palliative care services early in the illness trajectory can further support goal-concordant care planning.

## Discussion

FXTAS is a complex, progressive neurodegenerative condition that impacts not only the individuals diagnosed but also their families and caregivers. Characterized by a combination of motor dysfunction, cognitive decline, co-morbid psychiatric symptoms, and caregiver burden, FXTAS requires more than just disease-specific management. It demands a truly integrated, person-centered care model that evolves alongside the patient’s functional and emotional needs. See Table 5 for a clinical practice summary.

**Table 5:** Clinical practice summary: managing fragile X-associated tremor/ataxia syndrome (FXTAS).

1. Screening and diagnosis
Consider FXTAS diagnosis in adults over 50 with tremor, ataxia, cognitive decline, and/or other psychiatric symptoms.
Review family history for Fragile X-related conditions (FXS, FXPOI, FXAND).
Order *FMR1* CGG repeat testing to confirm premutation status.
Obtain MRI brain imaging, assessing for middle cerebellar peduncle (MCP) hyperintensities and WMH.
2. Early-stage management (Stages 1–3)
Refer for neurology consultation for motor symptom characterization and management.
Initiate balance and strength training with physical therapy.
Address psychiatric symptoms with psychiatric and clinical psychological evaluation.
Antidepressants for anxiety and/or depressive disorders.
Provide genetic counseling for patient and at-risk family members.
Connect families to Fragile X support organizations (e.g., NFXF).
3. Mid-stage management (Stages 3–4)
Begin occupational therapy for adaptive strategies and home safety assessments.
Refer for speech-language pathology evaluation for dysarthria, dysphagia, and cognitive- communication support.
Evaluate for hearing loss and refer to audiology as needed.
Introduce cognitive stimulation or digital brain training tools.
Discuss advance care planning (ACP) and document goals of care early
4. Late-stage management (Stages 5–6)
Shift focus to palliative care
Symptom management (e.g., pain, sleep, mood).
Swallowing safety and nutritional support.
Mobility support to prevent falls or injuries.
Avoid high-risk medications (e.g., benzodiazepines, anticholinergics, opioids) unless clinically necessary.
Coordinate home health, respite services, and hospice referrals when appropriate.
Support caregivers with mental health services, support groups, and respite care.
5. Caregiver and family support throughout
Provide ongoing education about disease progression and management strategies.
Address caregiver burden through counseling, peer support, and respite services.
Encourage participation in clinical research when available.
6. Key referral partners
Neurology
Psychiatry
Clinical Psychology
Speech-Language Pathology
Occupational therapy
Physical Therapy
Social Work/Case Management
Audiology
Palliative and Hospice Care Clinicians
Genetic Counseling

This paper outlines a comprehensive, multidisciplinary approach that bridges primary care, neurology, psychiatry, psychology, speech-language pathology, physical and occupational therapy, and social work. Such a coordinated care model helps both patients and caregivers to receive continuous support through all stages of the illness, from initial diagnosis through end-of-life care. Early interventions, including genetic counseling, mental health services, balance and mobility rehabilitation, and caregiver education, are critical in helping patients and caregivers to prepare for the challenges ahead.

As the disease progresses to more advanced stages, clinicians must shift their focus toward maximizing safety, preserving dignity, and ensuring comfort. This includes proactive fall prevention strategies, management of swallowing and communication difficulties, home modifications, and planning for progressive cognitive and behavioral changes. Care teams should engage families in advance care planning discussions early in the disease course, while patients still have decisional capacity, to ensure that medical interventions remain aligned with the individual’s goals and values.

In later stages, when patients may be requiring a wheelchair, bedridden, or experiencing profound cognitive impairment, a palliative approach becomes essential. Symptom management should prioritize relief from distressing physical and psychiatric symptoms while avoiding medications that pose unnecessary risks of sedation, delirium, or reduced quality of life. Coordination with hospice and palliative care providers can offer families additional support during this difficult phase, facilitating addressing both the patient’s and caregiver’s emotional, spiritual, and medical needs.

Ultimately, the goal of FXTAS care is not solely to manage symptoms but to optimize the quality of life for patients and their families across the disease continuum. By implementing a holistic, multidisciplinary model, clinicians can help reduce the burden of this under-recognized condition and empower families to navigate its challenges with knowledge, compassion, and resilience. Further research is needed to develop disease-modifying therapies and to expand evidence-based interventions that support long-term patient and caregiver well-being.

## Footnotes

1
www.uqora.com

2
https://prepareforyourcare.org

3
https://agingwithdignity.org>Programs>FiveWishes

4
https://theconversationproject.org
